# Modified intrascleral haptic fixation of the light adjustable lens in a case of spontaneous adult-onset bilateral lens subluxation

**DOI:** 10.1016/j.ajoc.2023.101864

**Published:** 2023-06-03

**Authors:** Chu Jian Ma, Craig C. Schallhorn, Jay M. Stewart, Julie M. Schallhorn

**Affiliations:** a Department of Ophthalmology, University of California, San Francisco, San Francisco, CA, USA; b Francis I. Proctor Foundation, University of California, San Francisco, San Francisco, CA, USA

**Keywords:** Light adjustable lens, Lens subluxation, Scleral-fixated intraocular lens, Yamane, Intrascleral haptic fixation

## Abstract

**Purpose:**

To describe the application of the light adjustable lens (LAL) using an intrascleral haptic fixation (ISHF) technique for the correction of aphakia and post-operative refractive error.

**Observation:**

The LAL was placed using a modified trocar-based ISHF technique for visual rehabilitation following removal of bilateral cataracts in a patient with ectopia lentis. She ultimately obtained an excellent refractive outcome after adjustment with micro-monovision.

**Conclusions and Importance:**

Secondary intraocular lens placement has a much higher risk of residual ametropia than traditional in-the-bag lens placement. The ISHF technique with the LAL presents a solution for eliminating postoperative refractive error in patients requiring scleral-fixated lenses.

## Financial support

The Department of Ophthalmology is supported by an unrestricted educational grant from Research to Prevent Blindness, as well as a departmental Core Grant for Vision Research, NIH-NEI EY002162.

## 1 Introduction

Many techniques exist to treat aphakia in the absence of capsular support, each bearing its own advantages and disadvantages.^1^ Since its introduction, intrascleral haptic fixation (ISHF) of a three-piece intraocular lens (IOL) has become favored by many surgeons.^2^ However, all techniques for non-capsular IOL placement share a common problem of decreased refractive predictability when compared to in-the-bag IOL placement.^3^, ^4^, ^5^ For scleral-fixated lenses, small changes in the placement of the sclerotomies directly affect the effective lens position and the refractive outcome.^4^

Although residual ametropia can be treated with laser vision correction,^6^^,^^7^ postoperative adjustment of the IOL power allows for an elegant solution. The only lens currently available for adjustment of IOL power after implantation is the RxSight Light Adjustable Lens (LAL, RxSight, Aliso Viejo, CA).^8^ This lens is approved for in-the-bag placement, but given its three-piece design we hypothesized that it would be amenable to scleral fixation by a modified ISHF technique.

## 2 Case report

A 53-year-old woman with a history of high myopia and bilateral superior lens subluxation presented with bilateral cortical and nuclear sclerotic cataracts with complaints of decreased vision over several months. Her medical history was unremarkable with no history of trauma or drug use, and she had previously undergone a normal genetic workup for Marfan's syndrome. She was first noted to have lens subluxation by ophthalmology at age 41, and had been getting routine refractions with optometry prior to that.

**Fig. 1:**
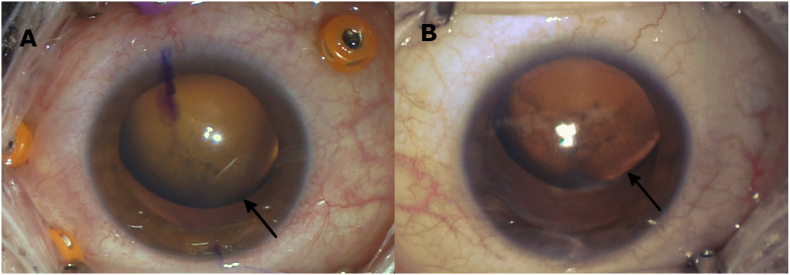
Intra-operative external photos of the right eye (A) and the left eye (B) with arrows indicating the equator of the dislocated crystalline lens.

On presentation, her best corrected visual acuity was 20/25 in the right eye and 20/30 in the left eye with a manifest refraction of −8.00 + 0.50 x 140 and -9.25 + 1.00 x 90, respectively. Ophthalmic examination of both eyes revealed superior subluxation of the crystalline lens with 1+ nuclear sclerotic and 3+ cortical cataracts (Fig. 1, right eye with mild subluxation, left eye with moderate subluxation). The fundus examination was within normal limits.

Given the degree of lens subluxation, the decision was made to remove the crystalline lens as well as the capsular bag and perform scleral fixation of an intraocular lens combined with complete pars plana vitrectomy. This approach allows for modified ISHF technique to reduce haptic manipulation. Upon discussion with the patient regarding IOL choices, she decided to pursue the LAL to allow for astigmatism correction and to minimize post-operative refractive error, understanding that it would be an off-label indication as the LAL is only approved for in-the-bag placement.

The patient's biometry (obtained from IOL Master 700, Zeiss) is presented in Table 1. The patient underwent sequential cataract surgery with pars plana vitrectomy, capsulectomy and scleral fixation of the IOL two weeks apart between the two eyes. +20.0 D LALs were selected with a target refraction using the Barrett Universal II formula of −0.13D for the right eye and +0.01D for the left eye. The technique used for modified scleral fixation of the lens has been previously described.^9^ Placement of a three-port 23-gauge trocar system was followed by marking and placement of two 27-gauge trocars at 12 and 6 o'clock, positioned 3 mm from the limbus and tunneled approximately 2 mm parallel to the limbus. Cataract extraction was done using a standard bimanual phacoemulsification technique. A vitrector was used to remove the bag complex and perform a pars plana vitrectomy with careful shaving of the vitreous base.

**Table 1:** Patient biometry data from IOL Master 700.

Eye	Axial Length (mm)	Anterior chamber depth (mm)	Steep meridian keratometry	Steep axisS	Flat meridian keratometry
**OD**	22.05	3.20	48.46	37	48.68
**OS**	21.93	3.37	48.22	8	49.48

**Fig. 2:**
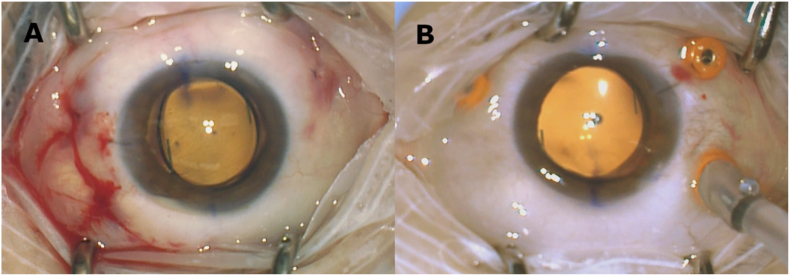
Intra-operative photos of the right eye (A) and the left eye (B) after scleral fixation of the lens.

The 3-piece LAL was then injected through the main wound and allowed to fall back onto the retina. The “Z” orientation of the lens was confirmed, and one of the haptics was then grasped at its end using 27-gauge forceps; the 27-gauge trocar cannula was moved up the shaft of the forceps, and the haptic was externalized and cauterized to form a flattened end. This was repeated with the second haptic. The lens was found to be in good position (Fig. 2).

The patient tolerated both surgeries without any complications and had a routine post-operative course. At time of first adjustment (about 5 weeks after the surgery for the right eye and three weeks for the left eye), the uncorrected visual acuity was 20/20 (right eye) and 20/50 (left eye). The patient's manifest refraction prior to any adjustments was +0.75 + 0.50 x 005 in the right eye, and +1.25 + 1.0 x 090 in the left eye. At that point, monovision was demonstrated to the patient in clinic, and she elected to pursue a mini-monovision approach. She underwent lens adjustment to target the right eye for −0.5 D and the left for plano. One month following her second lock-in treatment, her manifest refraction was −0.50 + 0.25 x 020 in the right eye and +0.50 + 0.25 x 150 in the left, with an uncorrected distance acuity of 20/20 in each eye and a near acuity of J3 in the right eye and J5 in the left (see Table 2 for details).

**Table 2:** Post operative refraction.

Timepoint	Right Eye	Left Eye
**POD1 UCVA**	20/60	20/20-
**POM1 UCVA**	20/20-	20/30, J3
**POM1 MRx**	+0.75 + 0.50 x5	+1.25 + 1.0- x 90
**Final UCVA**	20/20-; J3	20/20; J5
**Final MRx**	−0.5 + 0.75x20	+0.50 + 0.25 x150

UCVA – uncorrected visual acuity.

## 3 Discussion

There are several described techniques for the placement of an intraocular lens in the absence of capsular support, although they are all associated with high prediction error when compared to in-the-bag lenses and do not offer any method for astigmatic correction. The ISHF technique has been shown to result in 50% of patients within ±0.5 D of the intended target, versus 72% of patients within ±0.5 D for an in-the-bag IOL.^4^ The LAL, when used for in-the-bag placement can have up to 98% of patients within ±0.5 D^8^, which offers a significant advantage for the ISHF technique. Here, we present a novel application of a scleral-fixated LAL that allowed for correction of postoperative residual ametropia. We demonstrate that LAL allows for post-operative adjustments after scleral fixation similar to in-the-bag placement.

We employed the modified ISHF technique using 27-g trocars due to concern of haptic bending and manipulation that can occur with the traditional ISHF technique.^3^ The RxSight LAL uses a polymethylmethacrylate (PMMA) haptic, similar to that of the MA60AC lens (Alcon, Ft. Worth, TX) which can be susceptible to deformation or breakage during scleral fixation.^8^ The LAL requires excellent centration, as full exposure of the LAL optic to the UV adjustment light is essential for adjustment and lock-in. Any technique for ISHF of the LAL must be attentive to this goal.

The LAL is a viable lens for scleral fixation in patients with good preoperative dilation and a desire to minimize postoperative ametropia. This overcomes a major drawback of ISHF by allowing for post-surgical adjustment of any residual spherical or astigmatic refractive error. As an alternative to ISHF LAL placement, postoperative laser vision correction to correct residual ametropia is also a viable approach in patients who are good candidates for corneal refractive procedures.^6^^,^^7^ The pros and cons of each approach should be weighed when selecting the correct procedure for each patient.

There are some drawbacks to this case report. We cannot comment on postoperative lens tilt or lens position, both of which could affect refractive outcome.^4^ Additionally, patients undergoing scleral fixation of intraocular lenses have a higher risk of retinal detachment than the general population, possibly due to vitreous traction at the site of the sclerotomies.^10^ The potential for future retinal detachment repair and need for silicone oil placement should be a consideration when selecting which lens to implant as silicone IOLs have been shown to opacify in the presence of intraocular silicone oil.^11^ Likewise, steps should be taken during surgery to minimize entanglement of the vitreous and thus the potential for retinal tears when performing scleral fixation of a light adjustable lens. Further studies should be performed to assess the long-term positional stability and to investigate more fully the refractive outcomes of the LAL when used for scleral fixation.

## 4 Patient consent

Consent to publish the case report was not obtained. This report does not contain any personal information that could lead to the identification of the patient.

## Authorship

All authors attest that they meet the current ICMJE criteria for Authorship.

C.J. Ma: None.

C.S. Schallhorn: None.

J.M. Stewart: Merck (consultant), Genentech (consultant), Roche (other income), Long Bridge (Equity), Zeiss (consultant), Valitor (consultant).

J.M. Schallhorn: Zeiss (Consultant), Allergan (consultant), Forsight V6 (Consultant), Novus Vision (Consultant, Equity), Long Bridge (Consultant, Equity), Journey 1 (Consultant, Equity), Neurotrigger (Consultant, Equity).

## Declaration of competing interest

The authors declare that they have no known competing financial interests or personal relationships that could have appeared to influence the work reported in this paper.
